# Legal case documents: A comprehensive dataset for Arabic natural language processing research and applications

**DOI:** 10.1016/j.dib.2025.112429

**Published:** 2026-01-02

**Authors:** Soha Zarbah, Arwa Wali, Dimah Alahmadi

**Affiliations:** Information Systems Department, King Abdulaziz University, Jeddah, Saudi Arabia

**Keywords:** Legal case, Natural language processing, Arabic dataset, Artificial intelligence

## Abstract

The legal sector remains distinctive due to the complex language structure and specialized terminology of legal data. This complexity offers considerable contextual information, which demands natural language processing (NLP). The availability of high-quality and well-structured legal datasets is essential for advancing NLP research and applications within the legal field. However, a gap exists within the Arabic legal NLP owing to insufficient research and datasets. To address this gap, we aim to propose an Arabic legal case dataset containing cases, case summaries, relevant keywords, and case categories. The legal case data were obtained from the Board of Grievances website in Saudi Arabia and include 3170 cases distributed across 47 classes. The number of words in these cases varies significantly, ranging from about 100 to nearly 30,000 words per case. Moreover, the number of pages varies, ranging from one page to 80 pages per case. Therefore, this dataset supports various NLP applications, including text categorization, data extraction, sentiment analysis, and summarization, thereby improving task efficiency and decision accuracy in the legal profession.

Specifications Table

**Table:** 

Subject	Computer Sciences
Specific subject area	Machine learning, Transform learning, Natural language processing (NLP), Text summarization, Legal Arabic dataset.
Type of data	Table of pre-processed texts (.CSV file)
Data collection	The data was collected from published legal cases on the Board of Grievances website of Saudi Arabia. It contains 3170 cases distributed across 47 classes.
Data source location	The cases source is: https://www.bog.gov.sa/ScientificContent/JudicialBlogs/Pages/JudgmentsDefault.aspx
Data accessibility	The data are available freely for download Repository name: Kaggle Data identification number: https://doi.org/10.34740/KAGGLE/DSV/10529826 Direct URL to data: https://www.kaggle.com/datasets/sohaasiri/arabic-lega-case-dataset
Related research article	none

## 1 Value of the Data

• The dataset includes over 3000 Arabic legal cases, each with corresponding summaries, keywords, and categories.
• It contributes to Arabic NLP research by providing a curated legal dataset.
• All texts are written in Modern Standard Arabic, ensuring consistency and facilitating data analysis.
• The dataset’s reliability for NLP tasks was improved by removing errors, inconsistencies, and noise during the cleansing process.
• Case lengths range from 130 to 34,400 words, offering diverse examples that enhance NLP model training.
• The dataset is suitable for various NLP tasks, including machine translation, text classification, information extraction, summarization, and question answering.
• The dataset can be used to improve and test the transformer learning models, and it can be used with large language models (LLMs), such as GPT-4.

## 2 Background

While lexicons are being developed to understand and study Arabic dialects [1], the study of standard Arabic and the creation of lexicons that support it across various fields deserve greater attention. This paper addresses this issue by collecting a dataset specifically focused on the legal field, which could be utilized in various NLP tasks. Legal data are often complex owing to their language composition and specialized terminology, which provides considerable contextual information [2]. Consequently, the legal sector remains a distinctive and challenging field within natural language processing (NLP) [2]. High-quality and well-structured legal datasets are crucial for advancing in NLP research and applications, particularly in the context of the Arabic language [3].

Using NLP models in Arabic legal documents facilitates the extraction of important information from legal texts, the prediction of legal judgment, the identification of relationships among various components, and the summarization of essential concepts [[2], [3], [4], [5], [6]]. This assists legal professionals in case analysis, document review, and various legal research activities. Developing robust NLP models tailored to Arabic legal language can automate legal processes, enhance efficiency, and improve access to justice in Arabic-speaking nations [2]. Consequently, this study presents an Arabic legal case dataset (ALCD), which includes cases, case summaries, relevant keywords, and case categories [7]. The ALCD addresses a gap in Arabic legal datasets and facilitates the use of NLP techniques in this field.

## 3 Data Description

The ALCD was collected from the Board of Grievances (BoG) [8], a governmental body within the Saudi Arabian legal system [9]. This government website provides open data, including past case files in various categories adjudicated by the BoG. This data is accessible to all portal users, who can use it at their own risk. This right is guaranteed to all beneficiaries [10]. The adjudicated legal cases were in PDF format. Previously, BoG had jurisdiction over commercial and labor disputes, as well as objections against administrative decisions, and the domains focused on the collected cases. However, the BoG’s current jurisdiction only encompasses objections to administrative cases [9].

The collected cases were categorized into approximately 47 classes based on the classifications listed on the BoG website. The ALCD provides an organized and readily accessible source of Arabic legal data along with its related information, supporting various NLP applications, including text categorization, data extraction, sentiment analysis, and summarization. These applications can significantly enhance the efficiency and precision of legal tasks such as identifying case precedents, analyzing legal documents, and creating predictive legal models [11].

Although certain benefits are likely to be identified, the complexities of legal language and the subtleties of Arabic legal terminology require a comprehensive understanding of the dataset’s characteristics before beginning any NLP activity [11]. By analyzing the constructed dataset, we aim to establish a foundation for future NLP research. Specifically, it examined its structure, content, and quality.

Moreover, this study performed an exploratory data analysis to contribute to the advancement of NLP research in the legal field. This dataset provides valuable insights for scholars and practitioners aiming to use ALCD for Arabic NLP tasks.

## 4 Experimental Design, Materials, and Methods

**Fig. 1:**
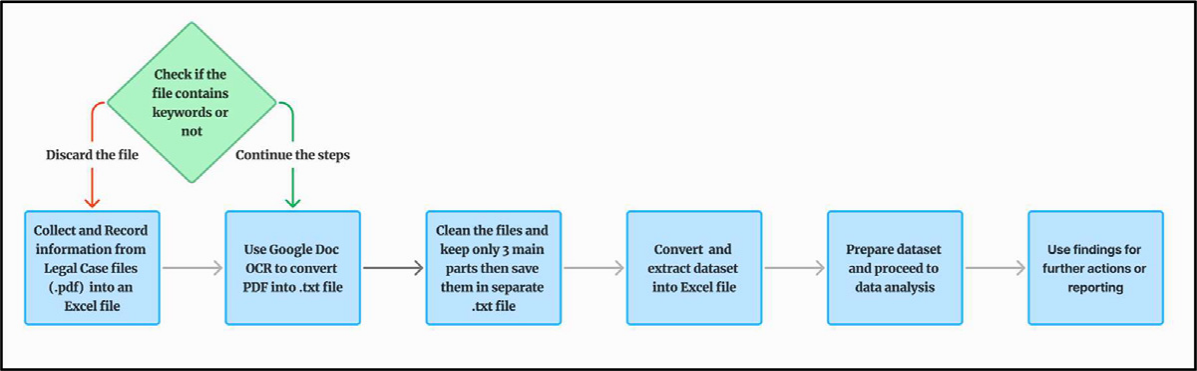
Arabic legal case dataset collecting steps.

The collection and analysis of the ALCD were conducted across various phases, as illustrated in Fig. 1. Each step is explained in detail in the following subsections.

### 4.1 Collect and record legal case files

**Fig. 2:**
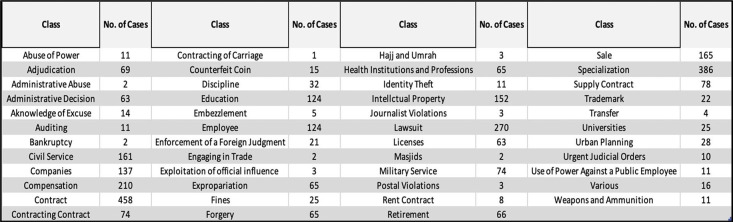
Number of cases for each class.

**Fig. 3:**
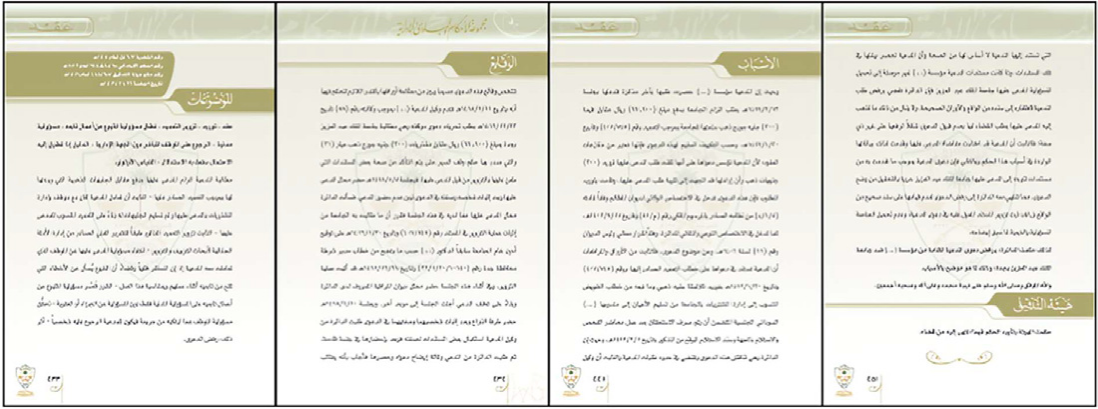
Arabic legal case PDF file example.

PDF files were downloaded from the BoG website [7]. As mentioned earlier, these files were categorized into 47 classes, where each class includes a set of cases as shown in Fig. 2. Subsequently, the necessary information, such as case classification, page count, word count per case, and other details, was recorded into an Excel file. Approximately 5000 case files were collected randomly and recorded across various categories. However, this number was constrained by time limitations, as the database is vital to other research within a defined timeframe. A key consideration was the inclusion of the keyword within the file. Files lacking these keywords were excluded from the analysis, thereby reducing the initial quantity of files from approximately 5000 to 3170. Examples of the contents of PDF files are illustrated in Fig. 3.

### 4.2 Convert PDF files to editable text files

PDFs were converted into text files using Google Docs' optical character recognition (OCR) feature. The outcome would represent a substantial advancement in text processing owing to its increased manageability [12]. The sanitized text files from the previous step included only case text, case summary, and keywords, excluding irrelevant information, such as case number and case date.

### 4.3 Cleaning and forming dataset folders

Each case was organized into a designated folder containing distinct text files: the case text file, which includes the entire case text; the case summary file; and the keywords file, which includes the keywords and topics related to the case.

### 4.4 Convert dataset folders into an Excel file

**Fig. 4:**
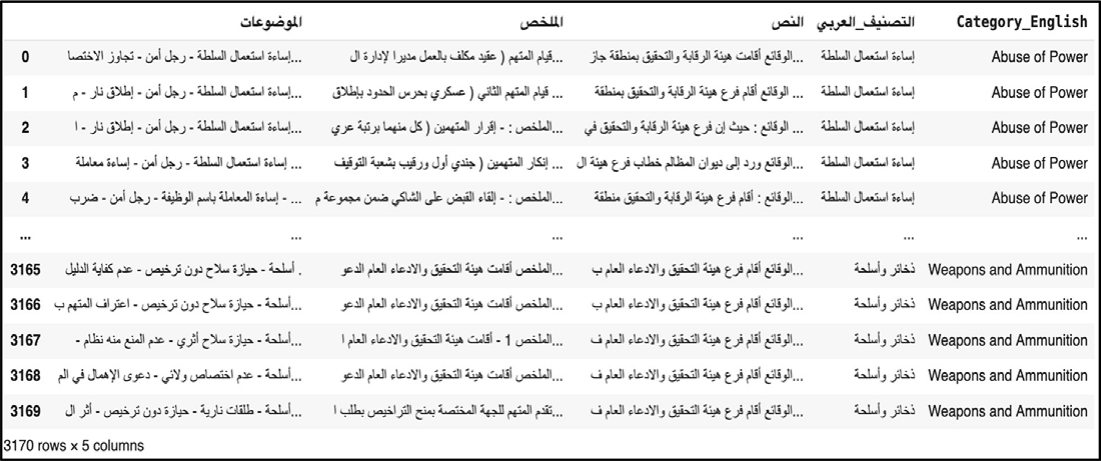
Dataset file format.

Upon completion of the collecting procedure, the folders and their contents were combined into a single Excel sheet, with each case represented in a single row and designated columns. The columns divide the legal case into three sections: case keywords, case summary, and case text. The remaining two columns indicate the category of the case in both Arabic and English, as shown in Fig. 4. This is because utilizing Excel files in Python facilitates the management of extensive datasets, execution of complex algorithms, and automation of repetitive activities [12].

### 4.5 Cleaning and preparing the dataset

Nonetheless, a comprehensive assessment of data quality was conducted despite these initiatives. All errors and inconsistencies in the data were identified and corrected using the state-of-the-art Arabic language model, AraBERTv2. This model addresses issues related to inconsistent extras and missing spacing. It standardizes characters and manages Arabic diacritics through a character encoding mechanism. Additionally, the AraBERTv2 model was employed to eliminate noise and standardize the text within the dataset [13]. Furthermore, the data were further processed using FarasaPy, a Python library specifically designed for segmenting Arabic text, to improve its quality [14]. This process resulted in a sanitized dataset suitable for analysis. Table 1 presents a sample text from a case before and after pre-processing. This example highlights multiple modifications implemented by AraBERTv2, including the removal of diacritics, the shortening of dots pointing to unknowns, the conversion of numerical language, the transformation of multi-paragraph text into a single paragraph, and the preservation of significant punctuation marks. A translation of the text content was included at the bottom of the table, and the symbol ``{…}'' indicates omitted sections. Generally, judicial statements are typically formal documents that require minimal processing [11], as demonstrated in the aforementioned example.

**Table 1:** Example of sample text from a case before and after pre-processing.

### 4.6 Dataset analysis

Data analysis revealed several key insights from the legal cases. The extracted knowledge can be utilized in multiple ways, such as generating reports, aiding juries in reaching accurate conclusions, developing NLP models, and applying it in legal research, particularly in summarization tasks. In this context, the ALCD dataset, which includes human-generated summaries for each case, distinguishes itself from other existing datasets.

This ALCD was produced using the aforementioned approach to ensure its dependability in diverse NLP applications and models. Table 2 presents summary statistics of the gathered legal dataset.

**Table 2:** Statistics of the gathered legal dataset.

Type of Information	Statistics
Number of cases	3170
Number of classes	47
Range of pages count for the cases	1 – 80
Average pages count	8
Maximum words count for the case	34,487
Minimum words count for the case	131
Average words count before pre-processing	1807
Average words count after pre-processing	1081

As mentioned, the initial step is to exclude cases that lack keywords before analysis. This step reduces the total cases from approximately 5000 to 3170, of which approximately 1830 cases lack keywords.

**Fig. 5:**
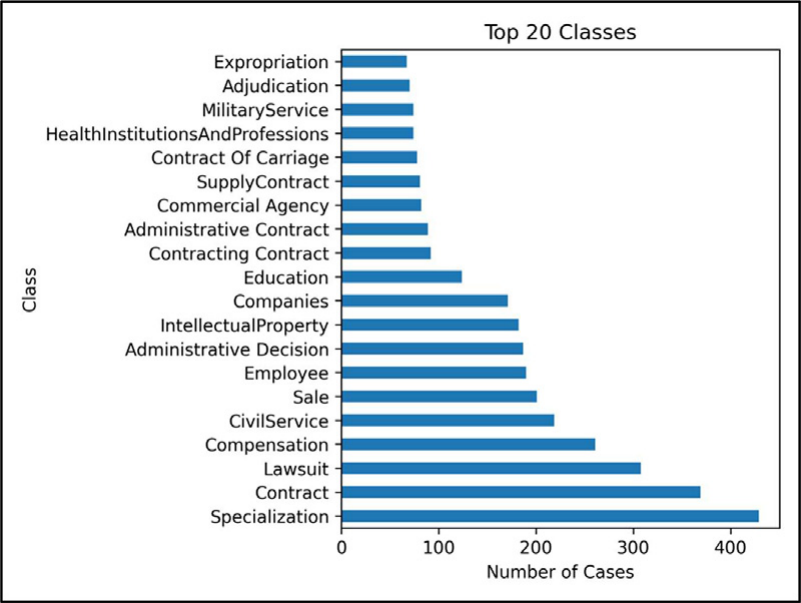
The highest 20 categories of the cases.

Table 2 indicates that there are 47 classes of cases, covering most of the classes on the BoG website; however, the number of cases within each class varies. Some classes contain >400 cases, while some contain only one or two cases as previously illustrated in Fig. 2. Fig. 5 shows the 20 categories with the highest case counts.

**Fig. 6:**
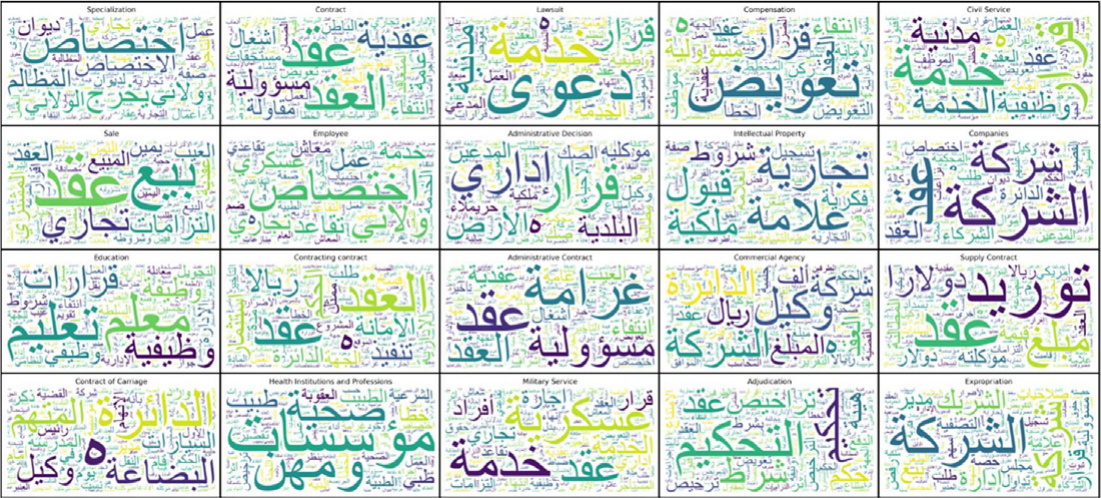
Word cloud of most used word according to the highest 20 categories.

**Fig. 7:**
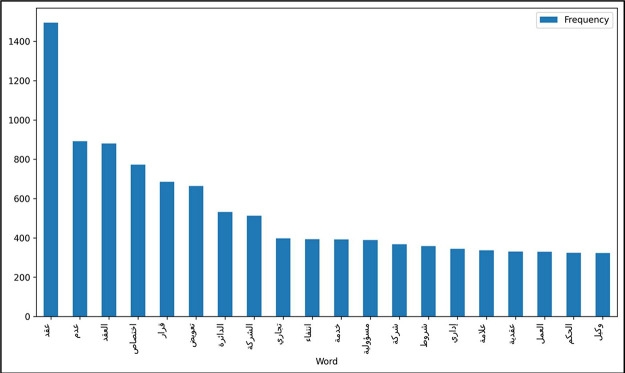
The 20 most frequently used words.

The textual contents of the dataset were examined, and the most commonly used words within each of the top 20 categories were displayed using a word cloud, as shown in Fig. 6. These word clouds clarify the most used words in cases. The order of most used classes begins from the top left corner with the term “specialization.” This is consistent across the remaining categories displayed in Fig. 5. Subsequently, the 20 most commonly used words in all cases, regardless of their classes, were extracted, as shown in Fig. 7. These words include contract, jurisdiction, non-existence, decision, compensation, the contract, department, absence, mark, trade, conditions, responsibility, administrative, contractual, acceptance, ownership, commercial, error, judgment, and service. These terms clarify the previously stated authority of the BoG, which included matters related to commerce, labor, and administration.

**Fig. 8:**
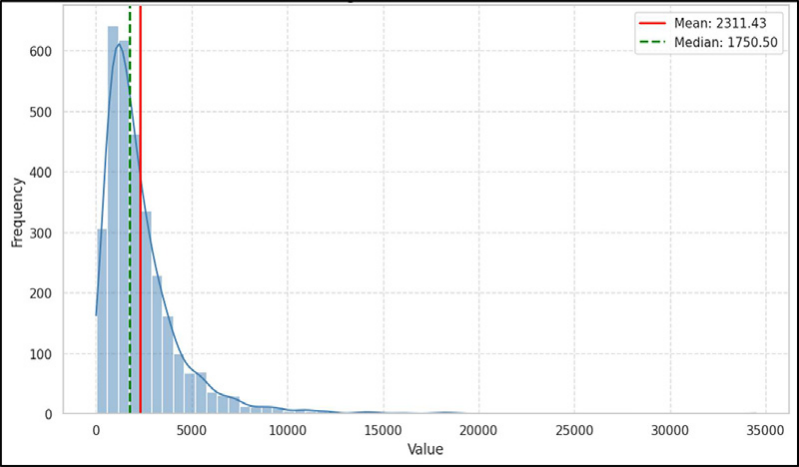
Histogram of full text's word distributions.

**Fig. 9:**
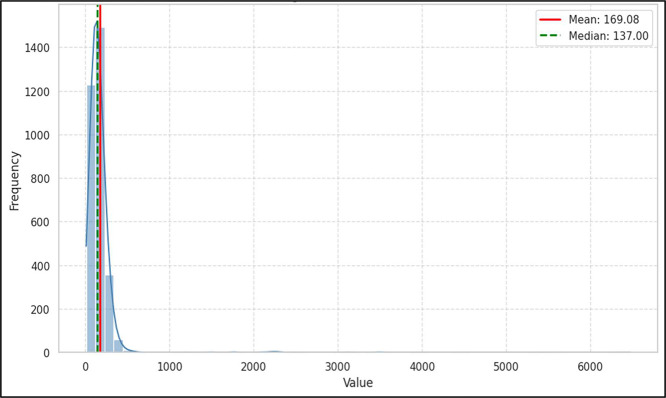
Histogram of summaries’ word distributions.

Additionally, the word count in a single case varies from 130 to over 34,000, as shown in Fig. 8, while the word count in a case summary ranges from approximately 50 to 6500, as illustrated in Fig. 9. Therefore, ALCD represents a stimulating resource for researchers interested in processing and analyzing long Arabic texts.

## Challenges during data collection

The challenges during the collection and preparation of data in our ALCD in relation to technological limitations, management of human resources, and scalability of processing the data include:

• Standard OCR tools face challenges concerning proper recognition of the Arabic characters, particularly in legal texts containing specialized terms and different font styles. Therefore, Google OCR was adopted due to its superior performance with Arabic text. Nonetheless, manual review remained necessary to correct errors during the conversion process.
• Most volunteers were from universities, which posed some challenges in terms of time availability and extracurricular activities.
• Training volunteers and paid assistants to categorize the data correctly was time-consuming and required continuous supervision to maintain quality and minimize errors.
• Converting the organized folders into Excel files was cumbersome, considering the gradual expansion of the dataset. The Excel files were also large to open directly without standard analysis tools.

These challenges highlight the difficulty in preparing a large-scale and high-quality dataset for most Arabic NLP tasks, a difficulty that is particularly pronounced for a language such as Arabic. Combining technical solutions with careful human oversight enabled us to develop a valuable resource for legal Arabic NLP research.

## Limitations

While this study resulted in a large ALCD, it has some limitations. The primary concern was the accuracy of several existing OCR systems, particularly in identifying dense Arabic texts and diverse font styles. To address this issue, manual review was necessary; however, it was time-consuming and susceptible to human error. Moreover, time constraints associated with the ALCD-related research deadline, along with the availability of volunteers and paid assistants, imposed limitations that necessitated ongoing training and supervision, ultimately reducing the number of cases in the collected dataset. These limitations highlight the complexities associated with constructing large, high-quality datasets for NLP tasks, particularly in languages such as Arabic, where unique linguistic characteristics and technical constraints may exacerbate the difficulties.

The ALCD dataset was collected due to the limitation of Arabic language data, especially in the legal domain. It opens up opportunities for various projects and research initiatives, such as the development and training of artificial intelligence models for NLP tasks in Arabic. These tasks include classification, judgment prediction, summarization, and more. Additionally, it facilitates the training of LLMs for a range of applications. These examples illustrate just a fraction of what the ALCD can contribute to the advancement of the Arabic language, particularly in its legal context.

## Ethics Statement

The authors have read and followed the ethical requirements for publication in Data in Brief and confirmed that the current work does not involve human subjects, animal experiments, or any data collected from social media platforms.

## CRediT Author Statement

**Soha Zarbah:** Conceptualization, Methodology, Software, Formal analysis, Investigation, Resources, Data curation, Writing—original draft preparation; **Arwa Wali:** Conceptualization, Methodology, Validation, Writing—review and editing, Supervision, project administration; **Demah Alahmadi:** Conceptualization, Methodology, Validation, Writing—review and editing, Supervision, project administration.

## Data Availability

KaggleArabic Lega Case Dataset (ALCD) (Original data).
